# Bypassing Pre‐Photoactivation in High‐Barrier Polyamide for Robust and Scalable Organic Persistent Luminescence

**DOI:** 10.1002/advs.76151

**Published:** 2026-07-01

**Authors:** Yunlong Yang, Sha Liu, Shilin Bo, Longming Jin, Baijun Tang, Zhiying Guo, Chengtao Gao, Xionggang Wang, Yuejun Liu, Qiang Zheng, Zi Liang Wu

**Affiliations:** ^1^ Hunan Engineering Laboratory of Polymer Packaging Materials Hunan Provincial Key Laboratory of Advanced Packaging Materials and Technology School of Packaging Engineering Hunan University of Technology Zhuzhou China; ^2^ Key Laboratory for Polymeric Composite and Functional Materials of the Ministry of Education School of Chemistry Sun Yat‐sen University Guangzhou China; ^3^ National Engineering Research Center for Compounding and Modification of Polymer Materials (Guizhou Material Industrial Technology Institute) Guiyang China; ^4^ Ministry of Education Key Laboratory of Macromolecular Synthesis and Functionalization Department of Polymer Science and Engineering Zhejiang University Hangzhou China

**Keywords:** industrial scalability, MXD6, organic persistent luminescence, oxygen/water barrier, without pre‐photoactivation

## Abstract

The critical trade‐off between ambient stability and moisture/oxygen barrier properties has long hindered the practical application of organic persistent luminescent (OPL) polymers, particularly for rapid‐response photoluminescence in demanding environments. Such limitations are manifested in direct‐doping systems, which suffer from humidity‐induced mechanical deterioration and sluggish excitation kinetics. Here, we overcome this fundamental limitation by molecularly locking chromophores within a poly(*m‐*xylene adipamide) (MXD6) matrix via melt doping. The engineered OPL composites leverage MXD6's exceptional barrier (oxygen transmission rate (OTR): 0.68 cm^3^·m^−2^·day^−1^·bar^−1^; water vapor transmission rate (WVTR): 5.3 g·m^−2^·day^−1^) to achieve efficient multicolor afterglow with high quantum yields (*Φ*_afterglow_ = 30.6%) and long lifetimes (5.6 s), operating stably without pre‐photoactivation. Remarkably, they retain >90% of afterglow efficiency after 30 days of continuous water immersion. Integrated experimental and computational studies reveal that multiple disordered hydrogen bonding simultaneously rigidify the polymer matrix and interlock chromophores, establishing an oxygen and water barrier for triple excitons. This strategy enables meter‐scale homogeneous fibers and transparent films that validate industrial viability for versatile luminescent devices. This work offers a universal paradigm for ambient‐stable OPL polymers, solving the stability‐barrier trade‐off to unlock rapid‐response luminescence in demanding environmental applications.

## Graphical abstract

A universal paradigm resolves the trade‐off between ambient stability and barrier properties in organic persistent luminescent (OPL) polymers. By interlocking chromophores within a high‐barrier MXD6 matrix, we achieved no Pre‐photoactivation OPLs with exceptional long‐term stability in water. Successfully fabricated on an industrial scale, these multicolor polymers are primed for versatile applications, including information encryption, displays, and high‐resolution 3D printing.

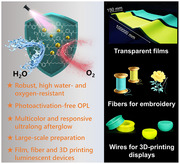

## 1 Introduction

Polymer‐based organic persistent luminescent (OPL) materials possess promising potential in anti‐counterfeiting [1, 2, 3, 4], dynamic displays [5, 6, 7, 8, 9], information storage [10, 11, 12], smart sensing [13, 14, 15], and bio‐imaging [16, 17, 18] applications. Compared to organic small‐molecule systems [19], these polymers exhibit superior mechanical flexibility, optical transparency, and ambient processability, enabling stable, tunable, and programmable luminescent functions. Among various fabrication routes, the direct‐doping strategy remains the most economically viable and industrially scalable approach. Embedding emitters within rigid polymer matrices through interactions such as hydrogen bonding [20], ionic bonding [21], or covalent cross‐linking [22] restricts molecular motion, enhances intersystem crossing (ISC), and establishes an oxygen‐diffusion barrier. This mechanism stabilizes triplet excitons, enabling rapid photoluminescence afterglow emission without the need for pre‐photoactivation. Polymers such as polyvinyl alcohol (PVA) [23, 24, 25, 26], polyacrylamide (PAM) [27, 28, 29, 30], and poly(acrylic acid) (PAA) [31, 32] serve as optimal matrices for this purpose. However, these materials face an intrinsic trade‐off: while structural rigidity effectively impedes oxygen diffusion for prolonged pre‐activation operation, the inherent hydrophilicity renders them vulnerable to moisture‐induced mechanical degradation. This mechanistic gap has limited their application in humid outdoor anti‐counterfeiting or biomedical environments.

Robust, water‐ and oxygen‐resistant polymer substrates offer pathways to overcome this trade‐off by simultaneously suppressing oxygen diffusion and mitigating moisture‐induced degradation. Cross‐linked polymers form highly dense 3D covalent networks that immobilize guest molecules and suppress triplet exciton quenching by oxygen. However, epoxy polymers [33, 34] and melamine‐formaldehyde [35] matrices exhibit limited flexibility due to restricted chain mobility from dense cross‐linking. Conversely, thermoplastic polymers, including poly(ethylene terephthalate) (PET) [35], polyethylene naphthalate (PEN) [36, 37], polylactic acid (PLA) [12, 38], polyamide 6 (PA6) [39], and recycled PET [40, 41] are attractive substrates due to their excellent flexibility and processability. Nevertheless, all these polymers suffer from undesirable oxygen permeability and solubility. Furthermore, the indispensable light‐activation process for achieving effective afterglow emission presents critical limitations, including excessive energy consumption, operational complexity, and slow response kinetics. These drawbacks inevitably hinder their use in rapid‐response or precision applications. Notably, the fabrication of OPL polymers that feature no prolonged pre‐activation operation, stability in harsh humid conditions, and mechanical flexibility via an industrially scalable route remains an unmet challenge.

Poly(*m*‐xylene adipamide) (MXD6), a semi‐aromatic polyamide synthesized from *m*‐xylenediamine and adipic acid, bridges the performance gap between aliphatic polyamides (PA6, PA66, and PA610) and fully aromatic aramids [42, 43]. Its rigid aromatic backbone, stabilized by interchain hydrogen bonds, creates a constrained microenvironment that immobilizes luminescent guests while simultaneously enhancing mechanical strength and oxygen barrier properties. Unlike high‐melting‐point aramids, MXD6 maintains facile melt‐processability, thus reconciling high‐performance barrier requirements with industrial‐scale manufacturing. Under standard measurement conditions (23°C, 60% RH), the oxygen transmission rate (OTR) of a 100‐µm‐thick film is 7.93 cm^3^·m∙^2^∙day^−1^bar^−1^ for PA6 and 11.59 cm^3^·m∙^2^∙day^−1^bar^−1^ for PET [44]; in contrast, a 20‐µm MXD6 film exhibits a significantly reduced OTR of 2.8 cm^3^·m∙^2^∙day^−1^bar^−1^ under identical test conditions [45], highlighting its exceptional barrier performance even at reduced thickness. The superior oxygen barrier properties of MXD6 protect oxygen‐sensitive triplet excitons, while its melt‐processability enables cost‐effective fabrication, rendering MXD6 an ideal matrix for rapid‐response OPL polymers. The large‐scale fabrication of luminescent materials offers substantial opportunities for practical applications [46]; and advanced processing techniques, such as 3D printing [38, 47, 48], facilitate customized device architectures. Overall, the unique combination of processability and barrier performance establishes MXD6 as a pivotal material for large‐area, customizable, and fast‐response OPL devices.

**SCHEME 1:**
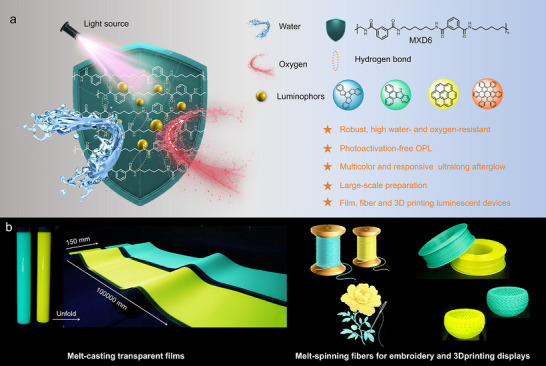
Schematic of rapid‐response multicolor OPL and associated potential applications. (a) Schematic representation of the mechanisms underlying efficient multicolor afterglow emission within the MXD6 matrix. (b) Diverse manufacturing modalities for OPL materials, highlighting their scalability and versatility: large‐area, flexible industrial‐scale homogeneous films; melt‐spun fibers for textile embroidery; and high‐resolution 3D‐printed architectures.

Here, we report a rapid‐response and water‐resistant organic persistent luminescent (OPL) polymer, composed of MXD6 matrix and luminophore, to achieve both satisfactory lifetime and efficient afterglow quantum yields (*Φ*_afterglow_) for large‐scale fabrication of commercially viable films and fibers. This melt‐doping strategy integrates chromophores into an interlocking microenvironment of hydrogen‐bonding networks within MXD6 chains. This structure confines molecular motion, enhances intersystem crossing (ISC), and establishes a robust oxygen/water barrier to protect triplet excitons. Using representative model compounds truxene (T) and 9H‐dibenzo[a,c]carbazole (D) as ultralong organic phosphorescence (UOP) emitters, alongside coronene (C) and hexabenzocoronene (H) as dual‐mode molecules exhibiting both UOP and thermally activated delayed fluorescence (TADF) [9, 37, 49], the obtained polymers exhibited broad afterglow emissions spanning blue to reddish orange, enabling a versatile multicolor palette (Scheme 1a). Notably, D@MXD6 and C@MXD6 exhibit *Φ*_afterglow_ of 30.6% and 22.3% with lifetimes of 4.5 and 5.6 s, respectively. Remarkably, after one month of aqueous immersion, D@MXD6 maintains robust afterglow performance with only a ∼10% reduction in *Φ*_afterglow_. Mechanistic investigations reveal that multiple disordered hydrogen bonding simultaneously rigidify the polymer matrix and interlocks chromophores, establishing an oxygen and water barrier for triple excitons. Lastly, we demonstrate that MXD6‐based OPL exhibits excellent processability, achieving industrial‐scale homogeneity in both melt‐spinning fibers (diameter: 100 µm; length > 500 m) and melt‐casting transparent films (100 m × 15 cm × 120 µm), enabling applications in information encryption, energy‐efficient displays, and high‐resolution 3D printing (Scheme 1b).

## 2 Results and Discussion

### 2.1 Materials Synthesis and Characterizations

**FIGURE 1:**
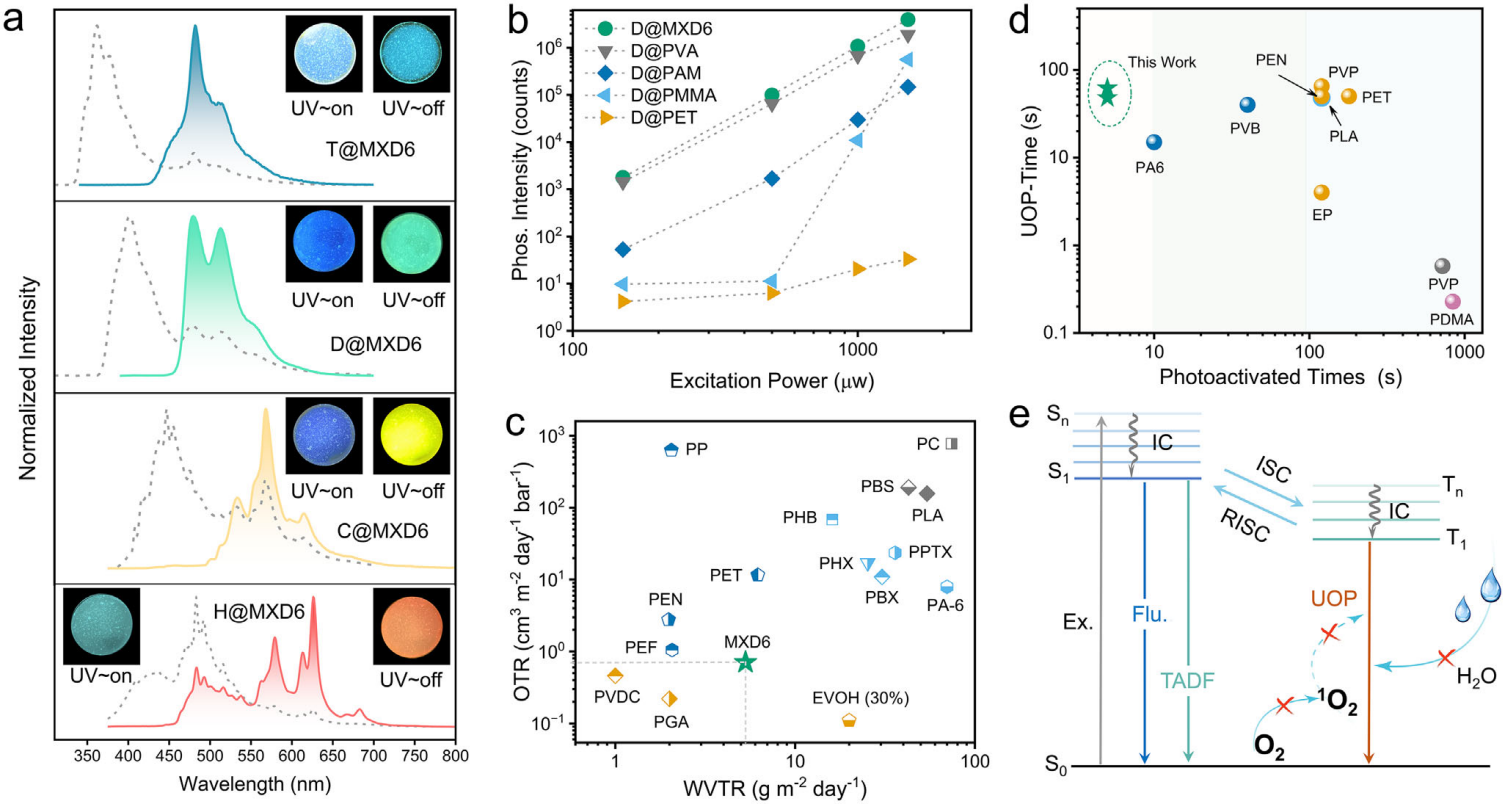
Material properties of MXD6 and UOP‐doped polymers. (a) Normalized steady‐state photoluminescence and delayed PL spectra for T@MXD6, D@MXD6, C@MXD6, and H@MXD6, dashed line: steady‐state PL. (b) Dependence of phosphorescence intensity on excitation power in D‐doped polymer matrices. (c) Water vapor transmission rate (WVTR; 37.8°C, 90% RH, 100 µm thickness) and oxygen transmission rate (OTR; 23°C, 60% RH, 1 bar, 100 µm thickness) of MXD6 compared with commercial polymeric materials [44]. (d) Relationship between UV irradiation time and afterglow duration of UOP in different polymer matrices. (e) A schematic illustration of the UOP mechanism, highlighting exceptional oxygen barrier properties and water resistance. Fluo. and Exc. refer to fluorescence and excitation.

To validate the advantages of MXD6 in fabricating organic persistent luminescent (OPL) materials, we prepared a series of multicolor‐emissive blend films by incorporating diverse chromophores through a melt‐doping process. The delayed photoluminescence (PL) spectra of T@MXD6, D@MXD6, C@MXD6, and H@MXD6 films exhibiting phosphorescence maxima at 480, 479 nm (with a shoulder at 513 nm), 567, and 625 nm, respectively. The corresponding Commission International de l'Eclairage (CIE) coordinate diagrams are located at (0.16, 0.33), (0.18, 0.47), (0.44, 0.53), and (0.44, 0.43), consistent with the naked eye observations (Figure 1a; Figure S1). To optimize performance, PL intensity and phosphorescence lifetime were evaluated as functions of dopant concentration, revealing optimal doping contents of 0.5 wt.% for T, D, and C@MXD6, and 0.05 wt.% for H@MXD6 (Figure S2). At these concentrations, the films achieve photoluminescence quantum yields (*Φ_PL_*) of 20.3%, 56.8%, 53.2%, and 19.9%, coupled with afterglow quantum yields (*Φ_afterglow_*) of 8.9%, 30.6%, 22.3%, and 10.2%, respectively (Figures S3 and S4a). The corresponding phosphorescence lifetimes are measured to be 2.1, 4.5, 5.6, and 3.0 s, respectively (Figure S4b). After removal of excitation with a UV‐light lamp, T@MXD6, D@MXD6, C@MXD6, and H@MXD6 exhibit remarkable blue, green, yellow, and reddish orange afterglows, lasting around 25, 48, 60, and 30 s, respectively, visible to the naked eye (Figure S5 and Videos S1–S4). Notably, the afterglow reached maximum duration after only a brief 5 s exposure to UV irradiation (10 mW cm^−2^), underscoring the rapid‐response characteristics of the MXD6‐based OPL materials. Additionally, the D@MXD6 and C@MXD6 films demonstrate outstanding *Φ_afterglow_* and a satisfactory lifetime among the recently reported polymer OPL materials [27].

To elucidate the mechanisms underlying rapid photo‐activation saturation, compound D was selected as a typical model due to its high *Φ_afterglow_* and long lifetime. In the D@MXD6 system, afterglow times remained consistent across UV irradiation intervals of 3, 5, 7, and 10 s (Video S5), indicating an exceptionally low activation threshold. In a further set of experiments, D was incorporated into diverse polymer matrices, including polyvinyl alcohol (PVA), polyacrylamide (PAM), polymethyl methacrylate (PMMA), and polyethylene terephthalate (PET). The obtained composites exhibited distinct trends in phosphorescence intensity as a function of excitation power (Figure 1b). Notably, under identical excitation conditions, D@MXD6 exhibited the highest phosphorescence intensity, followed by D@PVA and D@PAM in descending order. In contrast, the intensity for D@PMMA remained constant initially and then increased abruptly beyond a power threshold, while that of D@PET showed negligible change across the investigated power range (Figure S6). These trends are primarily attributed to the efficacy of photo‐induced oxygen consumption within each matrix. Oxygen serves as a potent triplet‐state (T_1_) quencher, destabilizing triplet excitons and suppressing phosphorescence. Under continuous UV excitation, ground‐state oxygen can be photosensitized to its singlet state oxygen (^1^O_2_) and subsequently consumed, thereby mitigating the quenching effect and stabilizing triplet excitons. The differential efficacy of this process across polymer hosts directly accounts for the observed variations in phosphorescence behavior. This result confirmed that MXD6 is an exceptional polymer matrix for enabling rapid‐response and stable OPL.

To quantitatively estimate oxygen and water barrier properties, oxygen transmission rates (OTR) and water vapour transmission rates (WVTR) were measured for both pristine and doped MXD6 films. Strikingly, the introduction of luminescent dopants did not compromise the barrier performance; both films maintained exceptionally low transmission rates, with OTR and WVTR values of approximately 0.64−0.67 cm^3^·m^−2^·day^−1^·bar^−1^ and 5.3 g·m^−2^·day^−1^, respectively (Figure 1c; Table S1). The remarkably low oxygen permeability of the amorphous MXD6 films stems from the rigid *m*‐xylylene units that suppress segmental chain motion, thereby constraining free‐volume pores to sizes below the kinetic diameter of oxygen (0.346 nm). This superior barrier performance is dominated by the synergistic effects of molecular rigidity and interchain hydrogen bonding. The reduced WVTR relative to PET arises from the dense hydrogen‐bonding network formed by amide groups, which establishes stable physical crosslinks that obstruct transient diffusion channels and resist water‐induced plasticization. Crucially, the hydrophobic benzene rings and the meta‐substitution geometry minimize moisture absorption, preventing humidity‐induced free‐volume expansion. Compared to other reported UOP polymer hosts, the excellent moisture and oxygen resistance of MXD6 not only enables the rapid phosphorescence response of the resulting UOP materials but also opens up possibilities for their use in real‐world environments (Figure 1d). Furthermore, the proposed luminescence mechanism for the MXD6‐based UOP materials is schematically illustrated in Figure 1e. The MXD6 matrix leverages a dense hydrogen‐bonded network and aromatic units to establish a rigidified microenvironment, which drastically mitigates vibrational dissipation. Beyond structural stabilization, its exceptional gas‐barrier properties isolate the vulnerable T_1_ excitons from atmospheric O_2_ and H_2_O quenching. When coupled with the TADF process, this robust encapsulation ensures both the high quantum yield and the persistent nature of UOP and TADF dual‐mode luminescence.

### 2.2 Evaluation of Environmental Stability

Environmental factors such as excitation light source, temperature, and humidity critically govern the performance of OPL materials. To assess the environmental stability of MXD6‐based OPL systems under practical conditions, we systematically characterized their excitation‐dependent phosphorescence profiles, thermally activated emission dynamics, and long‐term stability under high‐humidity conditions. We further explored the universality of MXD6 as a polymer matrix for high‐efficiency OPL by incorporating four representative luminophores at optimized concentrations: UOP phosphors (T and D) and dual‐mode UOP/ TADF emitters (C and H). Systematic characterization of the excitation‐wavelength‐dependent delayed photoluminescence (PL) spectra revealed that, with the exception of T@MXD6, all systems exhibited efficient UOP across a broad excitation range (300–420 nm). Notably, these systems displayed a prominent excitation peak at 360 nm and achieved remarkable broadband light‐harvesting capabilities (Figure S7), underscoring their potential for diverse photonic applications.

**FIGURE 2:**
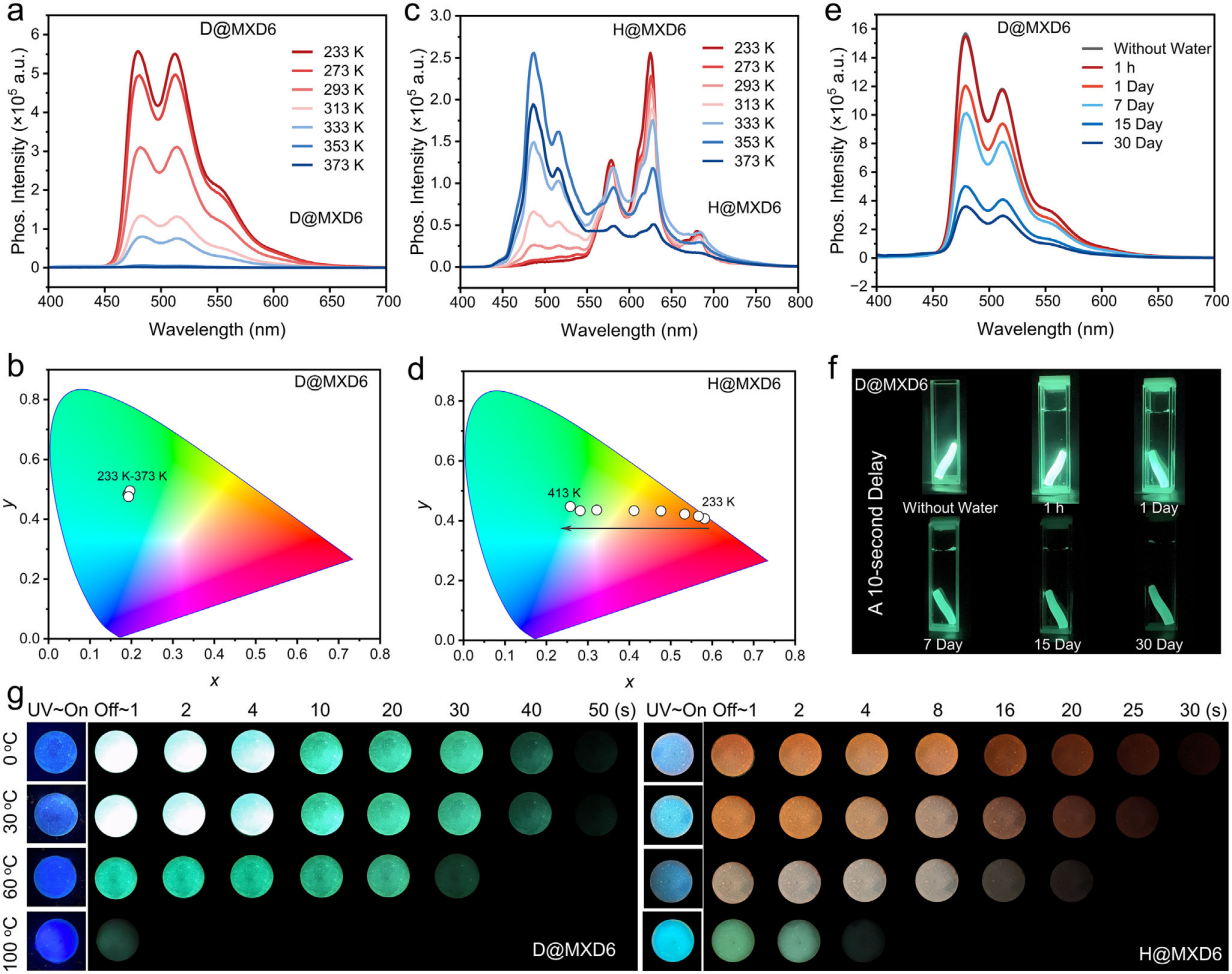
Photophysical characterizations of doped MXD6 UOP systems. (a) temperature‐dependent delayed PL spectra under 360 nm excitation, and (b) corresponding CIE chromaticity of D@MXD6. (c) Temperature‐dependent delayed PL spectra upon 360 nm excitation, and (d) corresponding CIE coordinates of H@MXD6. Evaluation of the stability for D@MXD6 under hydration conditions: (e) Delayed PL spectra and corresponding (f) afterglow images (captured 10 s after UV‐excitation cessation) of D@MXD6 under anhydrous conditions and after varying durations of aqueous immersion. Afterglow photographs of (g) D@MXD6 and H@MXD6 taken at various temperatures.

To elucidate the nature of delayed emission, temperature‐dependent phosphorescence spectra of D@MXD6 and H@MXD6 were analyzed under 360 nm excitation (233–373 K). For D@MXD6, the delayed emission intensity decreased steadily with increasing temperature (Figure 2a), confirming phosphorescent behavior and excluding TADF. Robust phosphorescence persisted up to the matrix's glass transition temperature (*T*_g_ ≈ 373 K), at which point the lifetime decreased sharply to 164 ms (Figure S8). This abrupt reduction arises from the matrix's glass transition, where segmental movement in amorphous regions of polymers destabilizes triplet excitons. Significantly, despite reduced phosphorescence intensity and lifetime at elevated temperatures, D@MXD6 maintained a distinct green afterglow lasting 20 s, with a lifetime of 2.05 s at 333 K (Figure 2b,g; Figure S8), highlighting its potential for elevated‐temperature applications. Unlike the phosphorescence‐dominated behavior of D@MXD6, H@MXD6 exhibited thermally responsive dual‐mode emission under identical excitation conditions [27, 37]. From 273 to 373 K, the intensity of the 483 nm delayed band increased concurrently with attenuation of the 627 nm band (Figure 2c), indicating distinct TADF (483 nm) and UOP (627 nm) characteristics. This spectral evolution was confirmed by dynamic shifts in CIE chromaticity coordinates from reddish orange to green upon heating, consistent with photographic observations (Figure 2d,g). Notably, H@MXD6 demonstrated a significantly longer phosphorescence lifetime than D@MXD6 at 373 K (Figure S9), attributable to its distinct molecular architecture and molecular interactions, aspects explored computationally below. The MXD6 polymer matrix consistently enables high‐temperature‐stable luminescence across diverse molecular systems, facilitating robust phosphorescence in D@MXD6 and thermally activated dual‐mode emission in H@MXD6 for practical applications under demanding thermal conditions.

We also explored the water‐oxygen barrier properties of the D@MXD6 polymers under harsh humid environments (Figure 2e,f). Following controlled aqueous immersion for varying durations, the phosphorescence emission intensity and afterglow duration were quantified. Unlike conventional doped systems (e.g., PAM and PVA), which suffer from rapid phosphorescence quenching upon exposure to moisture, the D@MXD6 composite displayed no discernible degradation in afterglow performance even after 30 days of continuous aqueous immersion. Notably, D@MXD6 exhibited a rapid photoluminescence response without the need for pre‐photoactivation across all immersion intervals. While the delayed‐PL intensity decreased from 16.0 ×10^5^ to 2.0 × 10^5^ (arbitrary units), the lifetime shortened from 4.5 to 2.9 s, while afterglow quantum yield (*Φ*_afterglow_) remarkably retained approximately 90% of its initial value (Figure S10 and Video S6). This combination of no prolonged pre‐activation operation and exceptional aqueous stability enables continuous functionality in high‐humidity environments.

### 2.3 Mechanism of the MXD6‐Based OPL

To gain deeper insight into the mechanism behind the efficient oxygen and water barrier properties of MXD6‐based doped systems, comprehensive molecular dynamics (MD) simulations were conducted on a system comprising 60 MXD6 and 10 O_2_ molecules. Following strict energy minimization and a 20 ns two‐stage dynamic relaxation, the system was subjected to a 10 000 ps isobaric–isothermal (NPT) simulation at 1 atm and 300 K, followed by a 10 000 ps constant‐volume (NVT) simulation (Figures S11a–d and S12a–c). The fully relaxed amorphous MXD6 thin‐film conformation reveals long‐range disordered molecular arrangements, wherein the molecular chains adopt distorted conformations. This enables the formation of an extensive, random, and irregular intermolecular hydrogen‐bonding network via interacting amide groups (–NH– and C = O) between adjacent polymer chains. Specifically, these N–H···O hydrogen bonds exhibit a compact average length of 2.15 Å, ranging tightly from 1.8 to 2.5 Å (Figure S11e, f). This simulated structural feature is further confirmed by Fourier‐transform infrared (FT‐IR) spectroscopy (Figure S13). MXD6 displays a broad, unstructured N–H stretching vibration peak centered near 3283 cm^−1^, corroborating the existence of multiple disordered intermolecular hydrogen bonds within the amorphous matrix. Such structural irregularity restricts chain segmental mobility, thereby suppressing crystallization and stabilizing the disordered amorphous state. Furthermore, this robust interchain network severely restricts local chain dynamics, endowing the MXD6 matrix with exceptional mechanical strength and thermal stability.

Crucially, this dense microenvironment inherently dictates the system's resistance to atmospheric quenching. The dynamic evolution associated with O_2_ permeation yields a restricted O_2_ diffusion coefficient of 7.28 × 10^−5^ cm^2^/s (Figure S12g), which is compounded by the formation of weak hydrogen bonds (1.283–1.296 Å) between the diffusing O_2_ and the MXD6 matrix. Consequently, in stark contrast to crystalline polymers that often suffer pronounced quenching in air, the delayed emission spectra and phosphorescence decay of the doped MXD6 system remain strikingly invariant under diverse atmospheric conditions. Together, these findings demonstrate that the disordered intermolecular hydrogen‐bonding network plays a profound dual role: rigidly inhibiting nonradiative decay pathways while kinetically and chemically suppressing oxygen diffusion.

**FIGURE 3:**
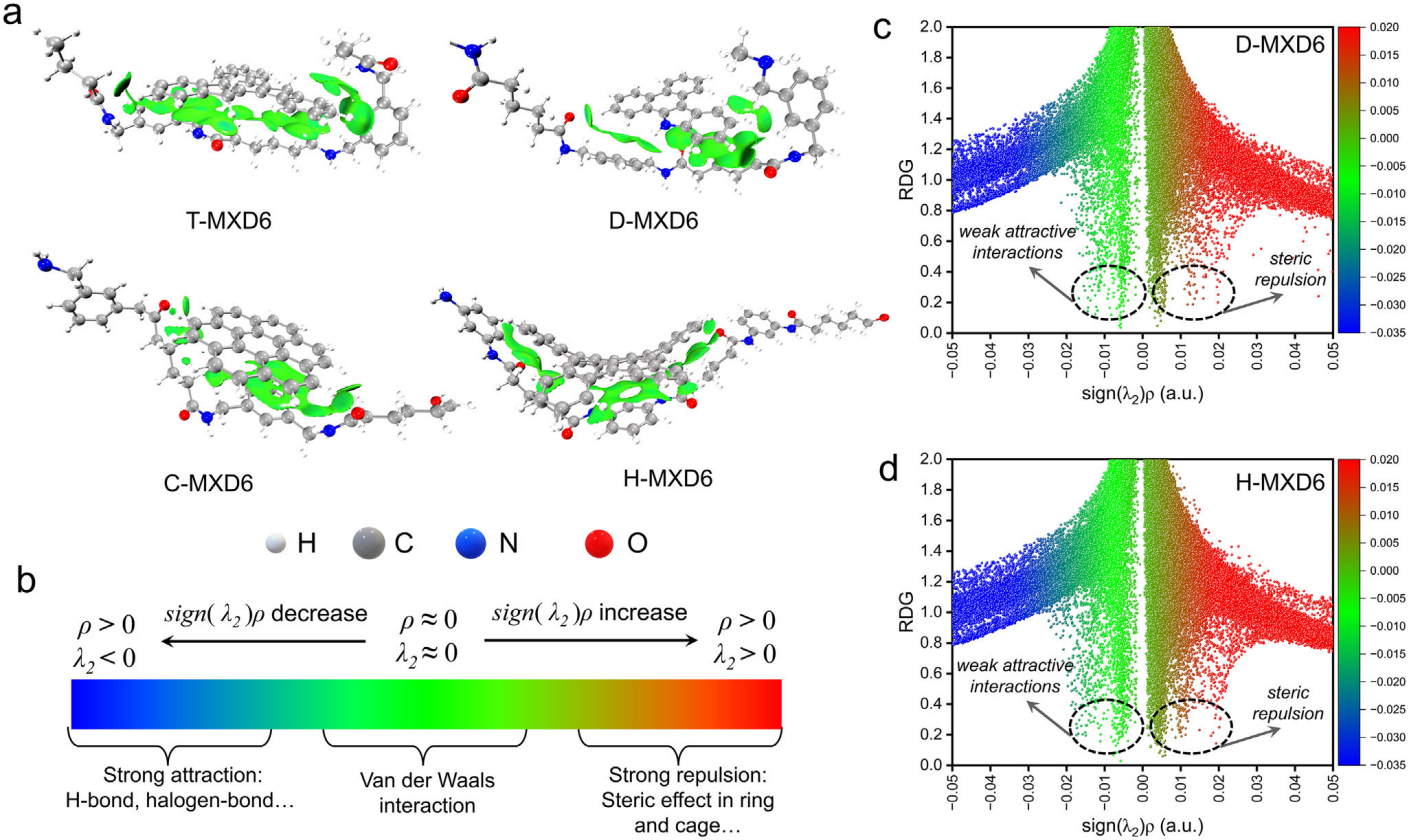
Analysis of the inter‐ and intramolecular interactions. (a) The visualization of intermolecular interactions of T‐MXD6, D‐MXD6, C‐MXD6, and H‐MXD6. (b) Common interpretation of the coloring method of the mapped function *sign(λ_2_)ρ* in IGM and IGMH maps. Scatter plots of RDG versus *sign(λ_2_)ρ* for (c) D‐MXD6 and (d) H‐MXD6.

To further elucidate how this disordered hydrogen‐bonding network governs the macroscopic barrier and photophysical properties, we employed the independent gradient model based on Hirshfeld partition (IGMH) to quantify the spatial distribution and strength of interchain non‐covalent interactions (NCIs). Building on the observation that the distorted conformations of MXD6 chains restrict segmental mobility, the IGMH analysis (Figure 3a,b) reveals a dense matrix of carbon–hydrogen–nitrogen contacts and aromatic hydrogen–nitrogen interactions. These short‐range interactions act as structural anchors that complement the primary N–H···O hydrogen‐bonding network (averaging 2.15 Å and ranging tightly from 1.8 to 2.5 Å) identified in the amorphous clusters. By providing this additional structural reinforcement, the molecular motion of the dopant systems is effectively constrained, thereby enhancing triplet exciton stability through the significant suppression of nonradiative dissipation pathways. The interplay between these cohesive forces and the localized structural environment was further visualized using reduced density gradient (RDG) plots (Figure 3c,d; Figure S14). The characteristic green isosurfaces, corresponding to the low‐electron‐density region (−0.02 ≤ *sign(λ_2_)ρ* ≤ 0.00), confirm the prevalence of intermolecular *π*–*π* stacking and van der Waals forces. This holistic balance between steric repulsion and weak attractive interactions validates the stability of the disordered amorphous matrix. Ultimately, this rigidified yet irregular molecular environment explains why the doped MXD6 systems maintain consistent phosphorescence decay and delayed emission across varying atmospheric conditions; the robust interchain network serves as both a physical barrier to oxygen quenching and a mechanical scaffold that inhibits the thermal vibrations typical of less constrained amorphous polymers.

The synergy between the rigidified MXD6 scaffold and the electronic landscape of the dopant molecules was further elucidated through electrostatic potential (ESP) surface analyses of the T/D/C/H‐MXD6 series. These analyses reveal distinct charge heterogeneities that critically govern their integration into the disordered MXD6 matrix. While T‐ and D‐MXD6 exhibit strong dipolar features that facilitate directional alignment with the polar amide groups of the polymer, C‐MXD6 possesses a prominent electron‐rich core, promoting the dominant *π*–*π* stacking interactions identified in the RDG analysis. In contrast, H‐MXD6 displays an alternating potential landscape, suggesting a more nuanced multi‐site interaction mode. These specific charge topologies drive the self‐assembly of the dopant systems through a coordinated interplay of electrostatic steering, van der Waals forces, and the aforementioned hydrogen‐bonding network. By acting as electronic and structural “plugs” within the amorphous voids, these dopants further reinforce the disordered intermolecular network, effectively sealing the matrix against oxygen permeation and suppressing the thermal vibrations of the polymer chains. Ultimately, these molecular‐level variations in ESP dictate the spatial packing modes, directly modulating the crystallinity, thermal stability, and the robust phosphorescence performance observed across the series (Figure S15). To investigate the excited‐state characteristics and spin‐orbit coupling (SOC) constants (ξ) of the luminophors, time‐dependent density functional theory (TD‐DFT) calculations were conducted. As shown in Figure S16, the UOP phosphors (T and D) and dual‐mode UOP/TADF emitters (C and H) exhibit appropriate energy separations, which better facilitate the fabrication of MXD6‐based OPL materials (Tables S2–S6).

### 2.4 Large‐Scale Fabrication and Thermal‐Mechanical‐Properties

**FIGURE 4:**
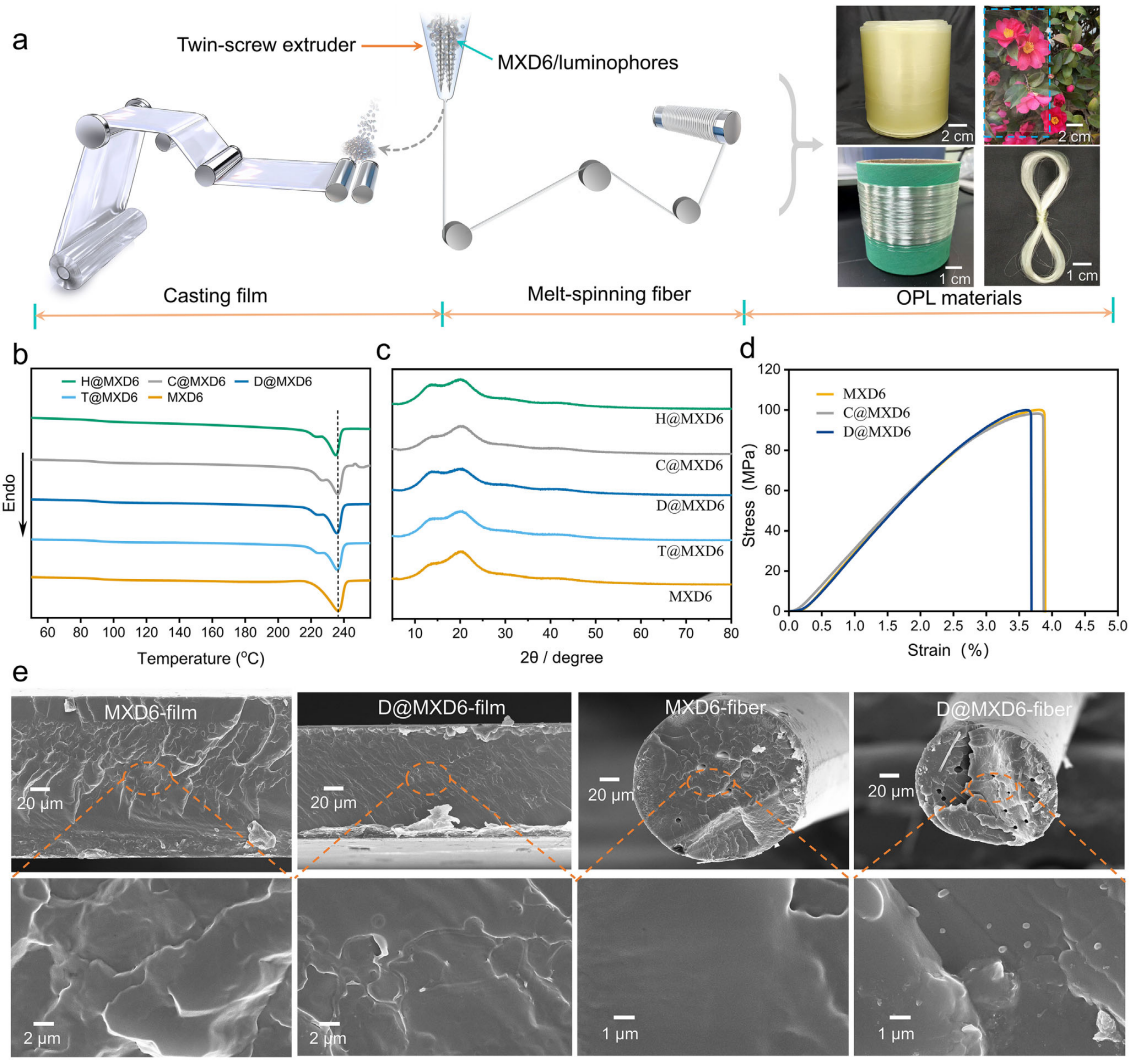
Large‐scale fabrication and material characterization of OPL platforms. (a) Schematic representation of the casting and melt‐spinning processes for doped MXD6, yielding large‐scale, high‐performance OPL materials. (b) Differential scanning calorimetry (DSC) thermograms of pristine MXD6 and the various doped emitters (T, D, C, and H). (c) X‐ray diffraction (XRD) patterns for pristine and guest‐doped MXD6, characterizing the crystalline phases. (d) Tensile stress–strain profiles of MXD6, D@MXD6, and C@MXD6 melt‐spinning fibers, highlighting their mechanical robustness. (e) Scanning electron microscopy (SEM) micrographs revealing the surface and cross‐sectional morphologies of MXD6 and D@MXD6 cast films and melt‐spun fibers.

Compared with previously reported polymer‐based OPL materials, the MXD6‐based OPL polymers offer a comprehensive improvement across four essential attributes: superior oxygen barrier performance, long‐term water stability, efficient afterglow luminescence without photo‐activation, and excellent melt‐processability. To demonstrate industrial scalability, we employed integrated twin‐screw extrusion and melt‐spinning to continuously produce OPL fibers with tunable diameters. The resulting chromophore‐doped MXD6 pellets were subsequently utilized in melt‐casting to fabricate large‐area transparent films (Figure 4a; Figure S17). We successfully produced T@MXD6, D@MXD6, C@MXD6, and H@MXD6 melt‐spun fibers (diameter: 100 µm; length > 500 m), which were then used for embroidering and knitting multicolored luminous textiles (Figure S18). Furthermore, OPL wires (diameter: 1.75 mm; length > 5 m) enabled the customized 3D printing of OPL devices (Figure S19), while melt‐cast transparent films (100 m × 15 cm × 120 µm) were applied in information encryption and energy‐efficient displays (Figure S20).

To evaluate the practical utility of the OPL polymers, we systematically characterized their thermal profiles. Assessment of thermal stability is fundamental to confirming the processing window and structural integrity of OPL systems under operational heat loads. Thermogravimetric analysis (TGA) revealed that the MXD6, T@MXD6, D@MXD6, C@MXD6, and H@MXD6 samples exhibited nearly equivalent decomposition temperatures (*T*_d_, 10%) of 391.5°C, 386.1°C, 387.0°C, 386.4°C, and 398.1°C, respectively (Figure S21). The negligible variance in (*T*_d_, 10%) across the doped series suggests that the incorporation of chromophores components does not compromise the inherent thermal stability of the MXD6 matrix. The differential scanning calorimetry (DSC) curve reveals a distinct glass transition temperature (*T_g_*) of 89°C, a melting point (*T_m_*) of 235°C for MXD6, T@MXD6, D@MXD6, C@MXD6, and H@MXD6 samples (Figure 4b). This thermal profile is a primary factor enabling MXD6 to maintain its superior oxygen barrier properties at temperatures below 90°C. To examine the influence of guest molecules on the internal microstructure of the polymer host, we performed wide‐angle X‐ray diffraction (XRD) measurements. The XRD patterns of the doped MXD6 systems remained identical to that of the pristine matrix, with no emergence of sharp crystalline reflections (Figure 4c). This confirms that guest incorporation occurs without inducing more phase separation or long‐range ordering. Consequently, the inherent amorphous‐dominant structural morphology of the MXD6 matrix is not significantly disrupted, demonstrating that the dopants are homogeneously distributed at the microscale without macro‐phase separation and do not act as nucleating agents that would alter the polymer's morphological state.

To further establish MXD6 as a scalable platform for OPL materials, quantitative assessments of mechanical strength and microstructural uniformity were conducted. Tensile testing demonstrated that dopant incorporation preserves mechanical properties comparable to the undoped polymer. Specifically, doped samples exhibited a tensile strength of 100 MPa, values that remain statistically consistent with the pristine polymer (Figure 4d; Figure S22). Scanning electron microscopy (SEM) revealed a uniform surface morphology with no discernible phase separation or dopant‐induced interfacial defects (Figure 4e). This lack of morphological deviation from the pristine matrix provides direct evidence of a homogeneous dopant distribution at the microscale. Ultimately, the preservation of mechanical integrity, coupled with the absence of phase separation, affirms doped MXD6 as a robust and homogeneous matrix suitable for industrial‐scale OPL production.

### 2.5 Potential Applications

**FIGURE 5:**
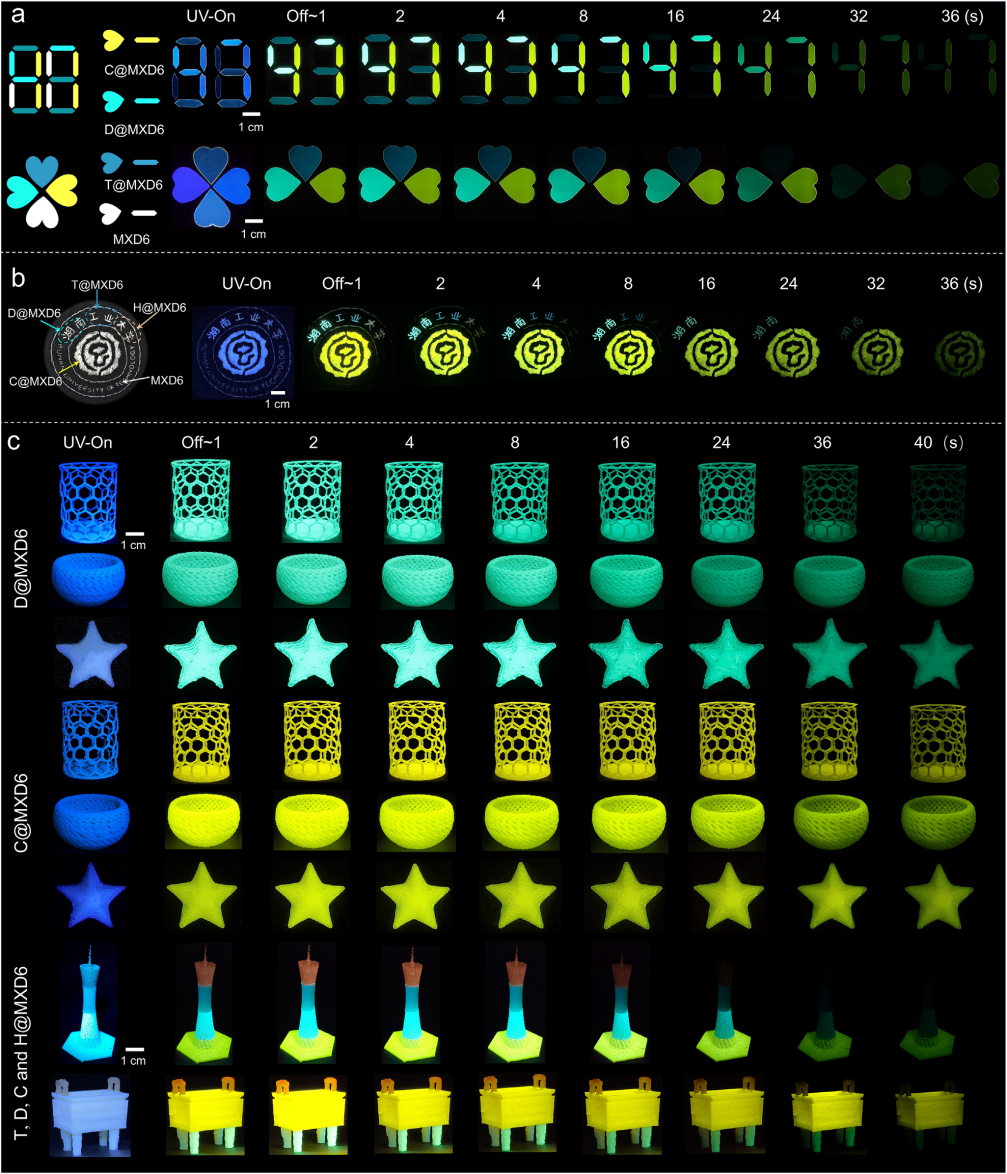
Description of OPL materials from casting‐films and spinning fibers enable high‐performance applications in persistent afterglow displays, advanced anti‐counterfeiting systems, and 3D‐printed architectures. (a) Transparent OPL patterns derived from cast films for high‐level optical encryption and authentication. (b) Embroidered motifs comprising OPL fibers with distinct emission colors enable spatiotemporally evolving afterglow displays; the time‐dependent intensity variations across discrete embroidered regions facilitate flexible, wearable long‐persistent architectures. (c) 3D‐printed architectures with varied porosities were fabricated using D@MXD6 and C@MXD6, monolithic devices were produced via the multi‐material integration of T@MXD6, D@MXD6, C@MXD6, and H@MXD6. Under excitation from a 365 nm source for a duration of 5 s, these structures demonstrate the rapid afterglow response dynamics inherent to the MXD6 matrix.

Leveraging the large‐scale fabrication of transparent films, we exploited the material's tunable color, long afterglow, and flexibility to create customized, complex luminescent patterns (e.g., gourd, butterfly; Figure S23). These patterns were utilized to demonstrate applications in time‐ and color‐dependent data encryption (Figure 5a; Video S7). Furthermore, by utilizing melt‐spun filaments with prolonged afterglow, we implemented embroidery of customized fiber patterns, enabling dynamic displays and showcasing the material's potential for flexible, wearable ultra‐long afterglow applications (Figure 5b; Video S8). Through melt‐extrusion, we successfully produced full‐color flexible fibers compatible with large‐scale 3D printing, confirming their excellent real‐time responsiveness. Capitalizing on the outstanding melt‐processability of the MXD6‐based oxygen‐barrier OPL material, we employed fused deposition modeling (FDM) 3D printing to fabricate diverse 3D objects (Figure S24), demonstrating its significant potential for large‐scale 3D printing applications. To validate the oxygen barrier properties, we printed 3D devices with controlled porosity (Figure 5c; Video S9). Under 365 nm UV irradiation, densely printed D@MXD6 and C@MXD6 objects achieved afterglow durations comparable to their low‐porosity counterparts, whether in porous or hollow 3D‐printed structures or in thick, solid 3D‐printed objects, the triplet excitons were well protected and not quenched. Enabling rapid response without the need for a pre‐photoactivation step, further confirming the material's high barrier efficacy against oxygen and water. This rapid‐response OPL material can be integrated into multifunctional devices; for instance, we demonstrated a 3D‐printed object exhibiting both time‐dependent decay and multi‐color afterglow, highlighting its versatility for large‐scale application in customized figurines, devices, and high‐level information encryption.

**FIGURE 6:**
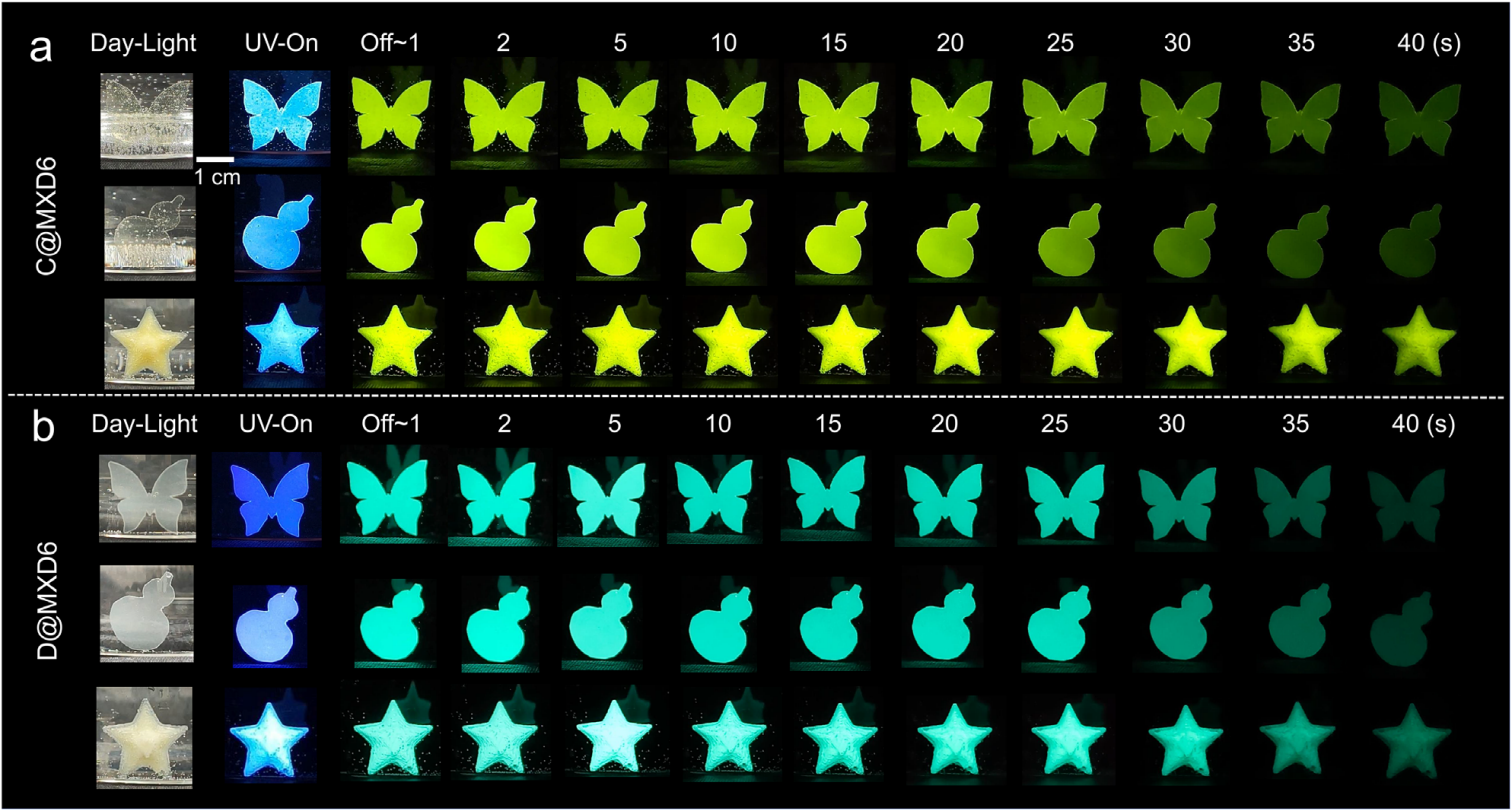
Underwater persistent luminescence and proof‐of‐concept applications. Time‐resolved optical photographs demonstrating the rapid activation and underwater persistent afterglow of (a) C@MXD6 and (b) D@MXD6 patterned films and 3D‐printed architectures. Both systems exhibit rapid‐response afterglow (up to 40 s) upon submersion without a prior photo‐charging process, highlighting the material's superior oxygen‐ and water‐barrier properties.

To further investigate the luminescent behavior of the fabricated films and corresponding 3D‐printed devices in aqueous environments, C@MXD6 and D@MXD6 patterned labels of various geometries were evaluated. Remarkably, these patterns achieved rapid activation in water without requiring a prolonged photo‐charging process, yielding a persistent afterglow of up to 40 s. Furthermore, the fabricated films maintained exceptional structural integrity and afterglow emission even after extensive aqueous soaking, highlighting the comprehensive stability of the material (Figures S25 and S26). The corresponding 3D‐printed architectures consistently maintained this 40 s underwater afterglow (Figure 6a,b), further verifying that the engineered OPL materials retain their rapid‐response persistent luminescence even when completely submerged. This robust aqueous stability establishes a novel material platform for advanced security and anti‐counterfeiting applications, such as underwater emergency signage. Building upon these findings, proof‐of‐concept underwater luminescent warning labels were successfully fabricated and demonstrated (Figure S27).

## 3 Conclusion

In summary, we report a universal paradigm for fabricating ambient‐stable, high‐performance, and multicolor organic persistent luminescent (OPL) polymers by leveraging the exceptional gas and moisture barrier properties of poly(*m*‐xylene adipamide) (MXD6). Through an industrially viable melt‐doping process, we achieved the molecular locking of diverse chromophores within the MXD6 matrix, yielding OPL composites with high afterglow quantum yields (up to 30.6%) and ultralong lifetimes (up to 5.6 s) without requiring pre‐photoactivation. Mechanistic analyses and simulations demonstrate that the rigid, semi‐aromatic backbone of MXD6 creates a dense interchain hydrogen‐bonded network that effectively suppresses triplet quenching. Notably, these materials retain >90% efficiency after thirty days of aqueous immersion, vastly outperforming traditional OPL hosts. The superior processability of this system, enabling large‐scale fibers, films, and 3D‐printed architectures− positions these OPL polymers as a robust platform for environmental‐stable organic phosphorescence in luminous textiles and photonic security applications.

## 4 Experimental Section/Methods

### 4.1 Preparation of the MXD6‐Based OPL Films

MXD6 pellets were subjected to vacuum desiccation at 80°C for 24 h to ensure the removal of residual moisture prior to thermal processing. The MXD6‐based OPL composites were fabricated via melt‐blending in a TDS‐35C twin‐screw extruder (Nanjing Corey Instruments, China). The luminophore was incorporated at a standardized concentration of 0.5 wt.%, unless otherwise specified. Extrusion was conducted with a screw speed of 180 rpm, utilizing a progressive thermal profile ranging from 150°C at the hopper to 190°C at the die. The resulting extrudate was water‐cooled and pelletized for subsequent film casting.

Large‐area transparent films (dimensions: 100 m × 15 cm) were manufactured using an FDHU‐35 extrusion casting system (Guangzhou Putong Instrument, China). Processing temperatures were maintained between 170°C and 210°C. Through precise modulation of the die head geometry and die lip aperture, the final film thickness was optimized to a uniform 120 µm. Custom patterns were subsequently created using embossing or laser‐engraving techniques.

### 4.2 Fabrication of MXD6‐Based OPL Fibers and Wires

Melt‐spinning was performed using a system (SHANGHAI CHANGKAI M&E Technology Co., Ltd) with MXD6‐based OPL pellets fed into auxiliary screws for melting, shearing, and plasticization. The resulting polymer melt was co‐extruded through precision spinneret orifices under regulated barometric pressure. Upon extrusion, the molten streams were quenched and solidified to yield a series of doped fibers. The fabrication process was maintained at a spinning temperature of 250°C with a take‐up velocity of 1200 m min^−1^. The resulting fibers exhibited high‐dimensional consistency, characterized by a diameter of 100 µm and continuous lengths exceeding 500 m. The OPL wires (diameter: 1.75 ± 0.03 mm) were prepared by feeding MXD6‐based OPL pellets into a miniature single‐screw extruder (SHANGHAI CHANGKAI M&E Technology Co., Ltd) under optimized conditions: barrel temperature of 250°C, screw speed of 50 rpm, and draw speed of 85 m min^−1^.

### 4.3 Production of 3D‐Printed OPL Devices

The 3D devices were fabricated with precisely controlled porosity and geometry using fused deposition modeling (FDM) on a Bambu Lab X1C printer (Shenzhen Bambu Lab Technology Co., Ltd.). Digital models were designed in 3Ds Max, converted to STL format, and wirelessly transmitted to the printer after slice processing. Printing was performed at a nozzle temperature of 260°C and a layer height of 0.18 mm. For hollow‐structured constructs, scaffold supports were added as necessary during slicing and removed post‐printing.

### 4.4 Computational Details

Amorphous MXD6 structures were constructed using the Amorphous Cell module in Materials Studio 2019, employing the Universal Force Field (UFF) to describe inter‐ and intramolecular interactions. Structural relaxation was performed via the Forcite module to yield a stable thin‐film conformation. Following equilibration, a 10 000 ps production simulation was conducted in the NVT ensemble to collect trajectory data. Representative conformations were subsequently extracted from the equilibrated system to serve as input for further analysis. IGMH analysis of weak interaction based on amorphous structure was conducted by using Multiwfn [50, 51]. The corresponding structure and IGM isosurfaces were generated using VMD 1.9.3 software [52].

## Funding

National Natural Science Foundation of China (Grant No. 12372245, 52325302), Natural Science Foundation of Hunan Province (Grants No. 2026JJ50490) and Hunan Provincial Education Department (Grant No. 24A0409), Guizhou Provincial Science and Technology Projects (No. [2024] 048) and Guizhou Provincial Major Scientific and Technological Program (No. [2024] 007).

## Conflicts of Interest

The authors declare no conflicts of interest.

## Supporting information




**Supporting File 1**: advs76151‐sup‐0001‐SuppMat.docx.

**Supporting File 2**: advs76151‐sup‐0002‐VideoS1.mp4.

**Supporting File 3**: advs76151‐sup‐0003‐VideoS2.mp4.

**Supporting File 4**: advs76151‐sup‐0004‐VideoS3.mp4.

**Supporting File 5**: advs76151‐sup‐0005‐VideoS4.mp4.

**Supporting File 6**: advs76151‐sup‐0006‐VideoS5.mp4.

**Supporting File 7**: advs76151‐sup‐0007‐VideoS6.mp4.

**Supporting File 8**: advs76151‐sup‐0008‐VideoS7.mp4.

**Supporting File 9**: advs76151‐sup‐0009‐VideoS8.mp4.

**Supporting File 10**: advs76151‐sup‐0010‐VideoS9.mp4.

## Data Availability

The data that support the findings of this study are available from the corresponding author upon reasonable request.
