# Supplementary material for: Bypassing Pre‐Photoactivation in High‐Barrier Polyamide for Robust and Scalable Organic Persistent Luminescence
Source: Adv Sci (Weinh). 2026 Jul 1;13(51):e76151. doi: 10.1002/advs.76151 (PMC13336969; doi:10.1002/advs.76151)
Supplement: Supplementary file 1 [file ADVS-13-e76151-s003.docx]

Supplementary Information

Bypassing Pre-photoactivation in High-Barrier Polyamide for Robust and Scalable Organic Persistent Luminescence

Yunlong Yang ^1^, Sha Liu ^1^, Shilin Bo ^2^, Longming Jin ^*2^, Baijun Tang ^1^, Zhiying Guo ^1^, Chengtao Gao ^3^, Xionggang Wang ^1^, Yuejun Liu^*1^, Qiang Zheng ^4^, Ziliang Wu^*4^

Y. Y., S. L., B. T., Z. G., X. W., and Y. L.

Hunan Engineering Laboratory of Polymer Packaging Materials, Hunan Provincial Key Laboratory of Advanced Packaging Materials and Technology, School of Packaging Engineering, Hunan University of Technology, Zhuzhou, 412000, China.

S.B. and L. J.

Key Laboratory for Polymeric Composite and Functional Materials of Ministry of Education, School of Chemistry, Sun Yat-sen University, Guangzhou, 510275, China.

C. G.

National Engineering Research Center for Compounding and Modification of Polymer Materials (Guizhou Material Industrial Technology Institute), Guiyang, 550014, China.

Q. Z. and Z. W.

National Engineering Research Center for Compounding and Modification of Polymer Materials (Guizhou Material Industrial Technology Institute), Guiyang, 550014, China.

*Corresponding authors. E-mail:

[jinlm5@mail2.sysu.edu.cn](mailto:jinlm5@mail2.sysu.edu.cn);

[yjliu_2005@126.com](mailto:yjliu_2005@126.com);

[wuziliang@zju.edu.cn](mailto:wuziliang@zju.edu.cn)

**Contents**

1. Additional experimental details	3

2. Photophysical properties	3

3. Applications	24

4. Supplementary Videos	25

5. References	25

1. Experimental details

**Materials**

Truxene (T, ≥98%, Aladdin Chemical Co. Ltd.), coronene (C, 98%, Bide Pharmatech),9H- hexabenzo[bc,ef,hi,kl,no,qr]coronene (H, 98%, Bide Pharmatech), dibenzo[a,c]carbazole (D, ≥98, Aladdin Chemical Co. Ltd.), Poly(hexamethylene isophthalamide) (MXD6, China Railway Rolling Stock Corporation Limited), toluene (AR, Macklin Ltd.), all chemicals were used as received without further purification.

**Measurements**

Oxygen barrier measurements were carried out at 23 °C and 60 % RH using a Brugger GTT gas transmission tester. Water vapor permeability tests were performed at 23 °C and 90 % RH using a Labthink C360M instrument. The tensile properties of the as-prepared samples were evaluated using a CMT4104 testing machine (SANS, China) at an extension rate of 20 mm min^-1^. Thermogravimetric analysis (TGA) was performed on a TA Q800 analyzer in a nitrogen atmosphere from 20 to 600 °C with a ramping rate of 10 °C min^-1^. Differential scanning calorimetry (DSC) was conducted using a NETZSCH DSC 300 Caliris (heating/cooling rate: 10 °C min^-1^ for second scan). Structural characterization employed a Bruker D8 ADVANCE XRD (2θ range: 5°–90°) and a Zeiss Supra 55 SEM. Optical properties were quantified via Shimadzu UV-2700 UV-vis spectroscopy and Edinburgh Instruments FLS1000 photoluminescence (PL) spectrophotometry, equipped with Xe900/μF900 excitation sources and an Oxford OptistatDN cryostat (77–500 K). Steady-state PL, time-resolved decay kinetics, absolute PL quantum yields (*Ф*_PL_), and delayed emission spectra were recorded on this system. Complementary *Ф*_PL_ measurements utilized a Hamamatsu Quantaurus-QY absolute PLQY spectrometer (*Φ* = 150 mm integrating sphere). Phosphorescence quantum yields were derived from the integrated intensity ratios of steady-state PL spectra via peak deconvolution analysis. Steady-state luminescence and afterglow images were documented using a Smartphone camera.

Water stability tests: samples were immersed in static, neutral deionized water (pH ≈ 7.0, 20–28 °C) for 30 days without stirring or water replacement. Long-term stability was evaluated by monitoring afterglow quantum yield retention and physical integrity. Negligible mass change (< 1%) and the absence of visible swelling confirmed the material’s robust resistance to water absorption and structural deformation. Post-immersion optical properties were re-characterized using the FLS1000 spectrophotometer.

TD-DFT calculations

The quantum chemistry calculation was performed on Gaussian 16 program.^[1]^ The ground states of the molecules were optimized by DFT calculation with B3LYP/CC-pVTZ basis set. ^[2,3]^ The excited states and SOCME investigation were calculated by TD-DFT calculation with B3LYP/CC-pVTZ basis set. Spin-orbit coupling (SOC) matrix elements were evaluated with PySOC,^[4]^ which called the MolSOC code to calculate the atomic integrals. Parameters for the effective charge in the operator for the atomic integrals were taken from the MolSOC code,^[5]^ without further optimization. Frontier molecular orbital (FMO) distributions and ionization potentials – derived from the energy differential between neutral and cationic species – were systematically characterized using the same theoretical framework. For comprehensive electronic structure interpretation, Multiwfn software processed wavefunction data to generate transition density matrixes and charge redistribution profiles, subsequently rendered as three-dimensional isosurfaces in Visual Molecular Dynamics (VMD) using particle-based volumetric representations. ^[6,7]^

2. Photophysical properties

**Figure S1.** CIE chromaticity coordinates of phosphorescence spectra for T@MXD6, D@MXD6, C@MXD6, and H@MXD6.

**Figure S2.** Steady-state photoluminescence, phosphorescence spectra, and lifetime decay profiles for phosphorescence emission in doped MXD6 films at various doping ratios. (a), (b) and (c) is the T@MXD6, excitation wavelength is 310 nm; (d), (e) and (f) is the D@MXD6, excitation wavelength is 360 nm; (g), (h) and (i) is the C@MXD6, excitation wavelength is 365 nm; (j), (k) and (l) is the H@MXD6, excitation wavelength is 365 nm.

**Figure S3.** The afterglow quantum yields of C@MXD6 calculated from the area ratios of steady-state and delayed spectra. Doping concentration: 0.05 wt%.

**Figure S4.** Ultralong organic phosphorescence performance of the doped films. (a) Quantum yields and (b) lifetime decay curves for T@MXD6, D@MXD6, C@MXD6, and H@MXD6.

**Figure S5.** Afterglow images of doped MXD6 films under flashlight excitation in air at room temperature, with T@MXD6 at 310 nm and D@MXD6, C@MXD6, H@MXD6 at 365 nm.

**Figure S6.** Phosphorescence intensity as a function of excitation power of (a) D@MXD6, (b) D@PVA, (c) D@PAM, (d) D@PMMA and (e) D@PET.

**Table S1.** WVTR and OTR Values of MXD6 and D@MXD6.

|  | WVTR 23 °C, 90 % RH  (g m^-2^ day^-1^) | OTR 23 °C, 60 % RH  (cm^3^ m^-2^ day^-1^ 0.1 bar^-1^) |
| --- | --- | --- |
| MXD6 | 5.31 | 0.6479 |
| D@MXD6 | 5.32 | 0.6713 |

**Figure S7.** Excitation- phosphorescence mapping of (a) T@MXD6, (b) D@MXD6, (c) C@MXD6 and (d) H@MXD6.

**Figure S8.** (a) Steady-state PL spectra of D@MXD6 film at various temperatures. (b) Transient PL decay profile of D@MXD6 film at various temperatures under excitation at 360 nm.

**Figure S9.** (a) Steady-state PL spectra of H@MXD6 film at various temperatures. (b) Lifetime of H@MXD6 film at 484 and 624 nm under 365 nm excitation at various temperatures. Transient PL decay profile of H@MXD6 film at various temperatures under excitation at 365 nm for (c) 484 nm and (d) 624 nm.

**Figure S10.** Time-dependent evolution of lifetime and quantum yield for D@MXD6 during continuous aqueous immersion.

**Figure S11** Molecular dynamics snapshot at 10000 ps. (a) Energy change curve of MXD6 (b) O_2_ at MXD6 with energy change curve (c) Temperature variation curve of MXD6 (d) O_2_ at MXD6 with temperature variation curve (e) Distribution of hydrogen bonds N-H···O (f) Hydrogen bond angle

**Figure S12** End-to-end distance, radius of gyration, and hydrogen bond length of MXD6. (a) Snapshot of MXD6 (b) Chain length of MXD6 (c) Radius of MXD6 (d) Number of MXD6 (e) hydrogen bond length of MXD6. (f) Molecular dynamics snapshot for O₂ diffusion in MXD6 and the weak hydrogen bond distance formed by O₂. (g) Diffusion curves and diffusion coefficient of oxygen in the interior.

**Figure S13.** Fourier-transform infrared (FT-IR) spectra of MXD6, D@MXD6 and H@MXD6 film.

**Figure S14.** Common interpretation of colouring method of mapped function *sign(λ_2_)ρ* in IGM and IGMH maps. Scatter plots of RDG versus sign(λ2)ρ for (a) T-MXD6 and (b) C-MXD6**.**

**Figure S15.** Electrostatic potential distribution analysis. T-MXD6, D-MXD6, C-MXD6, H-MXD6.

**Figure S16.** Energy level diagrams showing ISC pathways and corresponding spin-orbit coupling constants (ξ). (a) T. (b) D. (c) C. (d) H.

**Table S2.** Calculated energy levels and electronic transition configurations of singlet and triplet excited states for molecule T.

| Excited State | Energy Level (eV) | Transition Configuration | λ_cal_ |
| --- | --- | --- | --- |
| S_1_ | 3.8501 | H-1->L+1············49%  H->L··········49% | 322.03 nm |
| T_1_ | 2.7497 | H-2->L+3·········4%  H-1->L········43%  H->L+1············43% | 450.90 nm |
| T_2_ | 3.2552 | H-5->L+4··········2%  H-3->L+2········5%  H-2>L+1············5%  H-1>L+1·········35%  H->L·········35%  H->L+3·········6% | 380.88 nm |
| T_3_ | 3.2552 | H-5->L+5···········2%  H-4->L+2······3%  H-2->L···········5%  H-1->L·········35%  H-1->L+3·········6%  H->L+1·········35% | 380.88 nm |
| T_4_ | 3.7033 | H-1->L+1·········46%  H->L·········46% | 334.79 nm |
| T_5_ | 3.7867 | H-5->L+5·······4%  H-4->L+2··········6%  H-4->L+5·········3%  H-3->L+4·····3%  H-2->L······15%  H-2->L+5·······2%  H-1->L·······12%  H-1->L+3·······14%  H->L+1······12%  H->L+2·······3%  H->L+3·······3% | 327.42 nm |
| T_6_ | 3.7868 | H-5->L+4·········4%  H-4->L+4······3%  H-3->L+2·····6%  H-3->L+5········3%  H-2->L+1······15%  H-2->L+4·····2%  H-1->L+1······12%  H-1->L+2······3%  H-1->L+3·······3%  H->L·······12%  H->L+3·······14% | 327.41 nm |
| T_7_ | 3.9506 | H-7->L+1········2%  H-6->L···········2%  H-5->L+2········15%   H-4->L+1··········3%   H-4->L+5········16%  H-3->L········3%  H-3->L+4········16%  H-2->L+2········8%  H-2->L+3········5%  H-1->L········3%  H-1->L+5········3%  H-1->L+6········3%  H->L+1········2%  H->L+4········3%  H->L+7········3% | 313.83 nm |

**Table S3.** Calculated energy levels and electronic transition configurations of singlet and triplet excited states for molecule D.

| Excited State | Energy Level (eV) | Transition Configuration | λ_cal_ |
| --- | --- | --- | --- |
| S_1_ | 3.5877 | H-1->L········8%  H-1->L+1·······4%  H->L·······37%  H->L+1·······47% | 345.59 nm |
| T_1_ | 3.1303 | H-3->L+2·······2%  H-2->L+1·······4%  H-1->L············2%  H->L·······75%  H->L+1·······7% | 396.08 nm |
| T_2_ | 3.7095 | H-2->L+1·········16%  H-1->L········14%  H-1->L+1·······7%  H->L··············16%  H->L+1·········35%  H->L+2·········2% | 334.24 nm |
| T_3_ | 3.8925 | H-3->L······3%  H-2->L···········3%  H-2->L+1·····4%  H-1->L·····25%  H-1->L+1·····8%  H->L+1·····44% | 318.52 nm |
| T_4_ | 4.0388 | H-2->L·········9%  H-2->L+1·······4%  H-1->L·········41%  H-1->L+1·········23%  H->L+2·········8% | 306.98 nm |
| T_5_ | 4.2213 | H-3->L·······14%  H-3->L+3··············3%  H-2->L+1···········5%  H-2->L+3···········3%  H-1->L+1···········12%  H-1->L+3···········3%  H->L+2···········42%  H->L+3···········4% | 293.71 nm |
| T_6_ | 4.3547 | H-5->L+1···········3%  H-4->L··········2%  H-4->L+1···········3%  H-3->L···········4%  H-3->L+2···········3%  H-3->L+3···········4%  H-2->L···········30%  H-2->L+1···········9%  H-1->L···········6%  H-1->L+3···········4%  H->L+2···········2%  H->L+3···········15% | 284.71 nm |
| T_7_ | 4.6366 | H-4->L···········6%  H-4->L+1···········8%  H-2->L···········23%  H-2->L+4···········3%  H-1->L+1···········12%  H-1->L+2···········20%  H-1->L+3···········3%  H-1->L+4···········3%   H->L+2···········3%   H->L+3···········5%  H->L+4···········2% | 267.40 nm |

**Table S4.** Calculated energy levels and electronic transition configurations of singlet and triplet excited states for molecule C.

| Excited State | Energy Level (eV) | Transition Configuration | λ_cal_ |
| --- | --- | --- | --- |
| S_1_ | 3.2316 | H-1->L········50%  H->L+1·······50% | 383.71 nm |
| T_1_ | 2.3304 | H-5->L+5·······3%  H-4->L+4·······2%  H-3->L+3············2%  H-2->L+2············3%  H-1->L+2············46%  H->L············46% | 532.09 nm |
| T_2_ | 2.9429 | H-1->L·········17%  H-1->L+1········32%  H->L·······32%  H->L+1··············17% | 421.30 nm |
| T_3_ | 2.9429 | H-1->L·········32%  H-1->L+1········17%  H->L·······17%  H->L+1··············32% | 421.29 nm |
| T_4_ | 3.0792 | H-1->L·········49%  H->L+1·········49% | 402.65 nm |
| T_5_ | 3.5628 | H-5->L+1·······8%  H-4->L··············7%  H-3->L···········11%  H-2->L+1·······11%  H-1->L+2··············16%  H-1->L+3···········3%  H-1->L+4·······6%  H->L+2··············3%  H->L+3···········16%  H->L+4·······7% | 348.00 nm |
| T_6_ | 3.5628 | H-5->L·······8%  H-4->L+1··············7%  H-3->L+1···········11%  H-2->L·······11%  H-1->L+2··············3%  H-1->L+3···········16%  H-1->L+4·······7%  H->L+2··············16%  H->L+3···········3%  H->L+5·······6% | 347.99 nm |
| T_7_ | 3.7409 | H-3->L+1···········16%  H-2->L·······16%  H-1->L+3··············29%  H->L+2···········29% | 331.43 nm |

**Table S5.** Calculated energy levels and electronic transition configurations of singlet and triplet excited states for molecule H.

| Excited State | Energy Level (eV) | Transition Configuration | λ_cal_ |
| --- | --- | --- | --- |
| S_1_ | 2.6913 | H-1->L········46%  H->L+1·······52% | 460.74 nm |
| T_1_ | 1.8962 | H-2->L+2·······21%  H-1->L+1·······29%  H->L············44% | 653.93 nm |
| T_2_ | 2.2778 | H-2->L·········28%  H-2->L+2········4%  H-1->L+1·······2%  H->L··············3%  H->L+2·········53% | 544.33 nm |
| T_3_ | 2.3275 | H-2->L+1······35%  H-1->L···········4%  H-1->L+2·····50% | 532.69 nm |
| T_4_ | 2.5054 | H-1->L·········29%  H-1->L+2·······3%  H->L+1·········63% | 494.87 nm |
| T_5_ | 2.5195 | H-1->L+1·······50%  H->L··············40%  H->L+2···········4% | 492.10 nm |
| T_6_ | 2.6306 | H-1->L···········63%  H->L+1··········33% | 471.32 nm |
| T_7_ | 2.9416 | H-2->L···········60%  H->L+2···········35% | 421.48 nm |

**Table S6.** Calculated energy levels and electronic transition configurations of singlet and triplet excited states for molecule MXD6.

| Excited State | Energy Level (eV) | Transition Configuration | λ_cal_/nm |
| --- | --- | --- | --- |
| S_1_ | 4.3708 | H-8->L 61%  H-7->L 5%  H-5->L 32% | 283.67nm |
| T_1_ | 3.6719 | H-8->L 62%  H-7->L 5%  H-5->L 31% | 337.65 nm |
| T_2_ | 3.6960 | H-10->L+1 10%  H-10->L+3 2%  H-9->L+1 6%  H-9->L+3 22%  H-7->L+1 7%  H-7->L+3 6%  H-5->L+3 2%  H-4->L+1 10%  H-3->L+1 24%  H-2->L+1 7% | 335.45 nm |
| T_3_ | 3.7241 | H-6->L+4 8%  H-3->L+5 5%  H-2->L+2 4%  H-2->L+5 21%  H-1->L+2 7%  H-1->L+4 12%  H-1->L+5 10%  H->L+4 28%  H->L+5 3% | 332.93 nm |
| T_4_ | 3.8376 | H-6->L+2 43%  H-6->L+5 5%  H-3->L+2 2%  H-2->L+2 6%  H-1->L+2 18%  H-1->L+5 22%  H->L+2 18% | 323.08 nm |
| T_5_ | 4.5734 | H-6->L+2 7%  H-6->L+5 3%  H-3->L+5 3%  H-2->L+4 6%  H-2->L+5 7%  H-1->L+4 13%  H->L+2 16%  H->L+4 2%  H->L+5 37% | 271.10 nm |
| T_6_ | 4.5961 | H-10->L+1 3%  H-10->L+3 6%  H-9->L+1 14%  H-9->L+3 9%  H-7->L+1 10%  H-7->L+3 3%  H-5->L+1 3%  H-4->L+3 9%  H-3->L+3 30%  H-2->L+3 9% | 269.76 nm |
| T_7_ | 4.5998 | H-6->L+4 4%  H-3->L+4 3%  H-2->L+2 8%  H-2->L+4 5%  H-2->L+5 11%  H-1->L+2 7%  H-1->L+5 19%  H->L+4 33%  H->L+5 3% | 269.54 nm |

**Figure S17.** The photographs of the equipment and process for transparent cast films.

**Figure S18.** Product display of the OPL polymers. The photographs of D@MXD6 and C@MXD6 spinning fibers under sunlight, UV~on and UV~off.

**Figure S19.** The OPL wires (diameter: 1.75 ± 0.03 mm) for 3D printing deviced.

**Figure S20.** Product display of the OPL polymers. The photographs of D@MXD6 and C@MXD6 transparent cast films under sunlight, UV~on and UV~off.

**Figure S21.** Thermogravimetric analysis (TGA) of pristine MXD6 and the various doped emitters (T, D, C, and H).

**Figure S22.** Tensile stress–strain profiles of MXD6, D@MXD6, and H@MXD6 melt-casting films, highlighting their mechanical robustness.

3. Applications

**Figure S23.** The afterglow photographs of different shapes from D@MXD6 and C@MXD6 transparent cast films.

**Figure S24.** The afterglow photographs of 3D-printed architectures with varied porosities were fabricated using D@MXD6 and C@MXD6, under excitation from a 365 nm source for a duration of 5 s.

**Figure S25.** Tensile stress–strain profiles of D@MXD film with different soaked time in water highlighting their mechanical robustness.

**Figure 26**. Time-resolved optical afterglow photographs of D@MXD6 patterned films under different soaked time in water.

**Figure S27.** Practical demonstration of underwater luminescent warning labels, showcasing the potential of these OPL materials in advanced security, anti-counterfeiting, and emergency maritime signaling.

4. Supplementary Videos

The supplementary movies were recorded by a smart phone camera under dark environment, including nine supplementary movies from Video 1 to Video 9.

5. References

[1]	M. J. Frisch, G. W. Trucks, H. B. Schlegel, G. E. Scuseria, M. A. Robb, J. R. Cheeseman, G. Scalmani, V. Barone, G. A. Petersson, H. Nakatsuji, X. Li, M. Caricato, A. V. Marenich, J. Bloino, B. G. Janesko, R. Gomperts, B. Mennucci, H. P. Hratchian, J. V. Ortiz, A. F. Izmaylov, J. L. Sonnenberg, Williams, F. Ding, F. Lipparini, F. Egidi, J. Goings, B. Peng, A. Petrone, T. Henderson, D. Ranasinghe, V. G. Zakrzewski, J. Gao, N. Rega, G. Zheng, W. Liang, M. Hada, M. Ehara, K. Toyota, R. Fukuda, J. Hasegawa, M. Ishida, T. Nakajima, Y. Honda, O. Kitao, H. Nakai, T. Vreven, K. Throssell, J. A. Montgomery Jr., J. E. Peralta, F. Ogliaro, M. J. Bearpark, J. J. Heyd, E. N. Brothers, K. N. Kudin, V. N. Staroverov, T. A. Keith, R. Kobayashi, J. Normand, K. Raghavachari, A. P. Rendell, J. C. Burant, S. S. Iyengar, J. Tomasi, M. Cossi, J. M. Millam, M. Klene, C. Adamo, R. Cammi, J. W. Ochterski, R. L. Martin, K. Morokuma, O. Farkas, J. B. Foresman, D. J. Fox, Gaussian 16, Revision C.01; Gaussian, Inc.: Wallingford, CT, 2016.

[2] Thorn H. Dunning, Jr., “ Gaussian basis sets for use in correlated molecular calculations. I. The atoms boron through neon and hydrogen,” *Journal of Chemical Physics* 90 (1989): 1007-102343.

[3] Axel D. Becke, “ Density-functional thermochemistry. III. The role of exact exchange ”*Journal of Chemical Physics* 98 (1993): 5648-5652.

[4]	T. Lu, Q. Chen, “Independent Gradient Model Based on Hirshfeld Partition: A new method for visual study of interactions in chemical systems,” *Journal of Computational Chemistry* 43 (2022): 539-555..

[5] T. Lu, Q. Chen, “Multiwfn: A multifunctional wavefunction analyzer. ”*Journal of Computational Chemistry*, 33 (2012): 580-592.

[6] X. Gao, S. Bai, D. Fazzi, et al., “Evaluation of Spin-Orbit Couplings with Linear-Response Time-Dependent Density Functional Methods,” *Journal of Chemical Theory and Computation* 13 (2017): 515-524.

[7]	T. Lu, F. Chen, “Multiwfn: A Multifunctional Wavefunction Analyzer,” *Journal of Computational Chemistry* 33 (2012): 580-592.
